# Zebrafish RNase T2 genes and the evolution of secretory ribonucleases in animals

**DOI:** 10.1186/1471-2148-9-170

**Published:** 2009-07-20

**Authors:** Melissa S Hillwig, Ludmila Rizhsky, Ying Wang, Alisa Umanskaya, Jeffrey J Essner, Gustavo C MacIntosh

**Affiliations:** 1 Interdepartmental Genetics Graduate Program, Iowa State University, Ames, IA 50011, USA; 2 Department of Biochemistry, Biophysics and Molecular Biology, Iowa State University, Ames, IA 50011, USA; 3 Department of Genetics, Development and Cell Biology, Iowa State University, Ames, IA 50011, USA

## Abstract

**Background:**

Members of the Ribonuclease (RNase) T2 family are common models for enzymological studies, and their evolution has been well characterized in plants. This family of acidic RNases is widespread, with members in almost all organisms including plants, animals, fungi, bacteria and even some viruses. While several biological functions have been proposed for these enzymes in plants, their role in animals is unknown. Interestingly, in vertebrates most of the biological roles of plant RNase T2 proteins are carried out by members of a different family, RNase A. Still, RNase T2 proteins are conserved in these animals

**Results:**

As a first step to shed light on the role of animal RNase T2 enzymes, and to understand the evolution of these proteins while co-existing with the RNase A family, we characterized RNase Dre1 and RNase Dre2, the two RNase T2 genes present in the zebrafish (*Danio rerio*) genome. These genes are expressed in most tissues examined, including high expression in all stages of embryonic development, and their expression corresponds well with the presence of acidic RNase activities in every tissue analyzed. Embryo expression seems to be a conserved characteristic of members of this family, as other plant and animal RNase T2 genes show similar high expression during embryo development. While plant RNase T2 proteins and the vertebrate RNase A family show evidences of radiation and gene sorting, vertebrate RNase T2 proteins form a monophyletic group, but there is also another monophyletic group defining a fish-specific RNase T2 clade.

**Conclusion:**

Based on gene expression and phylogenetic analyses we propose that RNase T2 enzymes carry out a housekeeping function. This conserved biological role probably kept RNase T2 enzymes in animal genomes in spite of the presence of RNases A. A hypothetical role during embryo development is also discussed.

## Background

Ribonucleases (RNases) have long been used as biochemical models of enzymology and protein folding, and also as models for molecular phylogenetic and evolutionary analyses [1-3]. The RNase A and RNase T2 families are among those better characterized. The acidic ribonuclease RNase T2 was first purified from *Aspergillus oryzae *and characterized by Sato and Egami [4]. The RNase T2 superfamily is widespread [1], with members in almost all organisms analyzed to date, including bacteria, fungi, plants, animals and even viruses. RNase T2 enzymes are secreted RNases without base specificity, and they can degrade all types of single-stranded RNA [1]. Phylogenetic analysis of this family has been carried out extensively in plants, in particular in models of evolution of gametophytic self-incompatibility [5,6] because a subclass of the RNase T2 family, the S-RNases, is involved in this process. The T2 family has expanded and diversified in plants, and each angiosperm genome sequenced so far contains five or more genes belonging to this family (A. Meyer and G.C. MacIntosh, unpublished). These genes are classified as S-RNases or as S-like RNases, depending on whether they are involved in the self-incompatibility process or not [7]. A nutritional role as phosphate scavengers and defense roles as antibacterial, antifungal, or antiviral agents are among the functions proposed for S-like RNases [1,7].

In animals, the vertebrate-specific RNase A superfamily has been exhaustively studied [2]. RNase A enzymes are secreted proteins with pyrimidine base-specificity that can degrade any kind of single stranded RNA, and in some cases double stranded RNA [8]. This family has also been used in a variety of evolutionary studies, from mammalian and vertebrate phylogenetics [3,9] to analyses of evolution of novel gene functions after gene duplications [10,11]. Among the biological functions assigned to RNase A family members are nutrition, as a phosphate and nitrogen scavenger in the gut [12], and defense, due to antibacterial and antiviral properties [13,14]. These functions are similar to those assigned to RNase T2 members in plants. In addition, while some enzymatic differences exist between these two families, the main substrate seems to be similar.

The RNase T2 family has experienced a large expansion and diversification in plants; [5,6](Meyer A and MacIntosh GC, unpublished), and a parallel can be drawn to the RNase A family expansion in vertebrates [9,13]. In spite of these similarities, RNase A members have not been able to completely replace RNase T2 functions in vertebrates, since at least one gene belonging to the latter family has been found in each animal genome completely sequenced.

To gain insights on the evolution and coexistence of these RNase families we analyzed RNase T2 members found in the zebrafish (*Danio rerio*) genome. We chose this organism because its genome has been completely sequenced, and all developmental stages, from early embryo to adult, can be easily obtained. In addition, well-detailed analyses of zebrafish RNase A genes have been recently published [15-17]. Here we show that the zebrafish genome contains two RNase T2 genes. Expression of RNase T2 genes in all adult and embryo tissues suggests that this family of RNases have a housekeeping function, in contrast to the roles of RNase A, which are tissue- and stress-specific. In addition high RNase T2 embryo expression is conserved in various eukaryotes, both plant and animals, suggesting that an embryo specific function could also be important to maintain this family's presence in vertebrates even after RNase A genes appeared.

## Results

### RNase T2 enzymes are present in zebrafish

**Figure 1:**
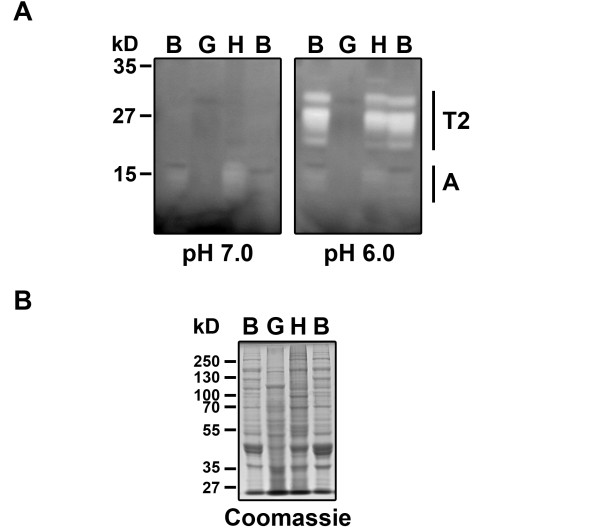
**Characterization of zebrafish RNases**. **A**) Ribonuclease activities present in zebrafish extracts. Adult zebrafish extracts were analyzed in an *in gel *RNase assay at two different pHs. Adult zebrafish of mixed sexes were separated into "body" (B, mostly muscle, skin and skeleton), "head" (H, which included skull, muscle, skin, brain, eyes among other tissues) and "gut" (G, which included most internal organs such as gut, liver, sexual organs, heart). The size range for RNase T2 and RNase A proteins is indicated. **B**) Same samples as in A, analyzed by SDS-PAGE and stained with Coomassie Blue. One hundred μg of protein per lane were analyzed in both types of gels.

Although early studies detected only faint RNase activity in fish organ extracts [18], an RNase T2 with acidic pH preference was recently isolated from salmon liver [19]. To identify RNase activities in zebrafish extracts we used a standard *in gel *activity assay that allows size separation of different proteins with RNase activity, as well as characterization of pH preference. Adult zebrafish of mixed sexes were separated into "body" (mostly muscle, skin and skeleton), "head" (which included skull, muscle, skin, brain, eyes among other tissues) and "guts" (which included most internal organs such as intestine, liver, heart, sexual organs). Crude extracts were then analyzed for RNase activities (Figure 1A). At neutral pH we identified only weak activities in the molecular weight range of RNase A (12–18 kDa). However, at an acidic pH we also observed stronger activities in the 20–30 kDa range that could correspond to RNase T2 enzymes. While body and head extracts clearly showed all activities, gut extracts did not show any detectable RNase activity in the conditions assayed. This result is not due to general protein degradation since protein integrity seems evident in a Coomassie stained SDS-PAGE (Figure 1B).

Identification of several bands in the RNase A range was consistent with the four RNase A genes found in the zebrafish genome [15,16]. Thus, several bands in the RNase T2 range suggested that the zebrafish genome also contained more than one RNase T2 gene. A BLASTP [20] search against the protein prediction database of the current zebrafish genome assembly (Zv7) using the RNase Ok2 sequence from salmon [19] identified two proteins with homology to RNase T2 enzymes, one located in chromosome 15 and the other in chromosome 13. Additional searches using these two proteins (using TBLASTN), or the corresponding nucleotide sequences (using BLASTN) against the full genome assembly and available ESTs failed to identify any additional sequences corresponding to RNase T2 homologs.

**Figure 2:**
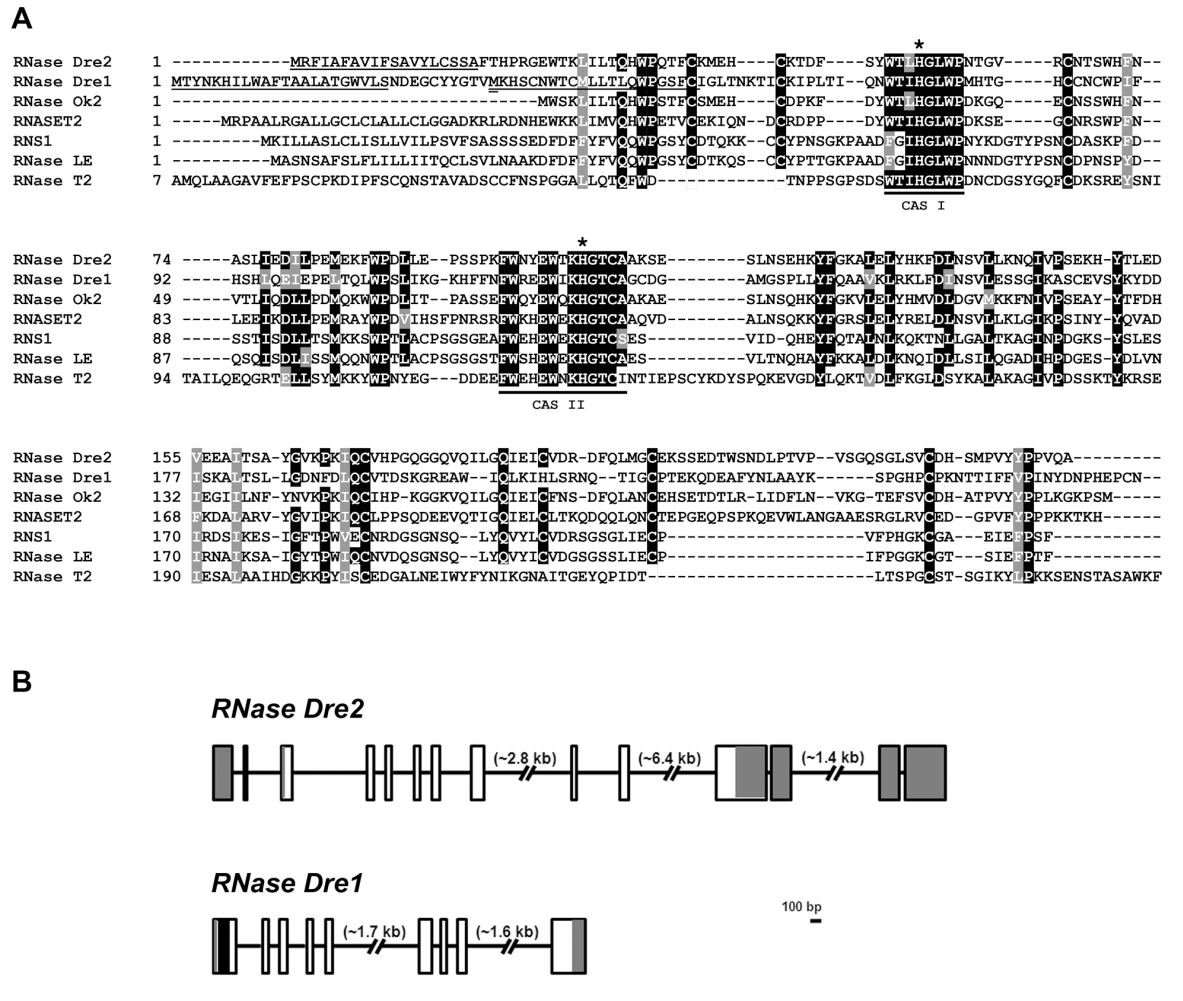
**The zebrafish genome contains two RNase T2 genes**. **A**) Alignment of the two predicted RNase T2 proteins (RNase Dre1 and RNase Dre2) present in the zebrafish genome with RNase T2 proteins from salmon (RNase Ok2), human (RNASET2), *Arabidopsis thaliana *(RNS1), tomato (RNase LE) and *Aspergillus oryzae *(RNase T2). Residues conserved in all RNase T2 enzymes are highlighted. CAS I and CAS II, conserved active-site segments that contain the two Histidines (*) involved in catalysis. The predicted signal peptides for RNase Dre1 and RNase DRe2 are underlined, and the alternative starting Methionine in RNase Dre1 is double-underlined. **B**) Structure of the two RNase T2 genes identified in the zebrafish genome. The intron-exon structure was obtained by comparison of the sequences obtained from direct cloning and sequencing of cDNA with the publish sequence of genomic DNA. Boxes indicate exons, lines indicate introns. Gray shading indicates untranslated regions, white indicates coding region, and black marks the region that undergoes alternative splicing in *RNase Dre1*. Gene accession numbers for the zebrafish proteins are FJ460212 for RNase Dre2 and FJ460210 and FJ460211 for the two different splicing variants of RNase Dre1.

The two proteins contain conserved amino acid sequences (CAS I and CAS II, Figure 2A) characteristic of the RNase T2 family, which include the His residues (* in Figure 2) essential for RNase activity. They also have conserved Cys residues important for establishment of tertiary structure, and other residues conserved in most RNase T2 homologs [1]. We named these enzymes RNase Dre1 (chromosome 15) and RNase Dre2 (chromosome 13). Molecular weights of the predicted mature peptides are 23.7 kDa and 27.3 kDa for the two forms of RNase Dre1 (see below) and 25.4 kDa for RNase Dre2. In addition, four N-glycosylation sites are predicted for RNase Dre1 and two for RNase Dre2. Incomplete glycosylation of the proteins at these sites could account for all the bands in the 20–30 kDa range observed in the activity gels shown in Figure 1.

Using RT-PCR we cloned cDNAs corresponding to both genes, and confirmed the sequence of the predicted proteins. Both predicted proteins appear to have signal peptides that may direct them to the secretory pathway. Based on the sequence of the predicted mature peptides, the identity between RNase Dre1 and RNase Dre2 is only 31%; in fact RNase Dre2 is more similar to RNASET2, the human RNase T2 homolog (44% identity), while RNase Dre1 is only 26% identical to RNASET2. On the other hand, both proteins are 22% (RNase Dre2) and 19% (RNase Dre1) identical to RNase T2 from *Aspergillus oryzae*, the prototypic RNase of the family[1]. Analysis of genomic organization (Figure 2B) showed that *RNase Dre1 *has 9 exons, while *RNase Dre2 *has 14, although the coding regions for both genes are contained in 9 exons.

During cloning we observed that *RNase Dre1 *RT-PCR showed two different bands, with less than 100 bp difference in size. Cloning and sequencing of individual bands (See Additional File 1) resulted in the identification of an alternative splicing variant. The presence of the alternative exon of 74 nt in the mRNA (black box in Figure 2B) results in a longer mRNA. The expression of both mRNA species was confirmed by Northern blots (not shown). This extra exon also results in a change in the open reading frame, which in turns changes the start codon position. Thus, the short mRNA species produces a longer peptide, while the long mRNA produces a shorter one. This change only affects the signal peptide (Figure 2A). Subcellular localization prediction programs predict that both isoforms of RNase Dre1, as well as RNase Dre2, contain a signal peptide that target the proteins to the secretory pathway, as is the norm for most members of the RNase T2 family. However, the putative signal peptide in the shorter RNase Dre1 protein includes sequences that are highly conserved in all RNase T2 proteins, suggesting that this putative peptide might not be cleaved, or that it could result in a non-functional protein.

### *RNase Dre1 *and *RNase Dre2 *are expressed in adult and embryo tissues

**Figure 3:**
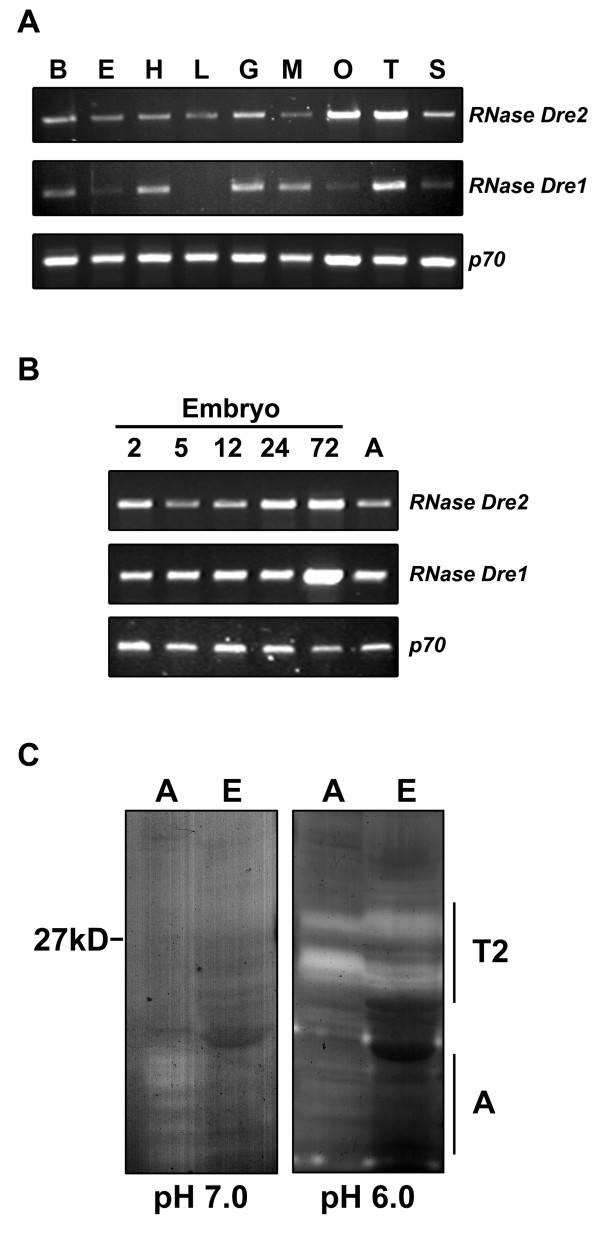
**Expression of zebrafish *RNase Dre1 *and *RNase Dre2***. **A**) RT-PCR analysis of expression of *RNase Dre2 *and *RNase Dre1 *in adult tissues: B, brain; E, eye; H, heart; L, liver; G, gut; M, muscle; O, ovary; T, testis; S, skin. p70 was used as control for loading. **B**) RT-PCR analysis of expression of *RNase Dre2 *and *RNase Dre1 *in embryos at different times (in days) after fertilization. **C**) Ribonuclease activities present in zebrafish embryos (E) and adults (A) analyzed by *in gel *activity assay as in Figure 1.

To determine when and where the two RNase T2 genes were expressed, we used semi-quantitative RT-PCR. Adult fish tissues were dissected and total RNA was isolated. To be able to compare *RNase Dre1 *and *RNase Dre2 *expression with that of RNase A genes, we used the same tissues utilized in the analysis by Cho and Zhang [16]. These included brain, eye, heart, liver, gut, muscle, ovary, testis and skin. *RNase Dre2 *was expressed in all organs, and a stronger signal was detected in reproductive organs (Figure 3A). *RNase Dre1 *was not detected in liver and the signal was weak in eye, ovary and skin under the conditions of our experiment. These expression patterns contrast with those observed for RNase A homologs, which are expressed almost exclusively in liver and gut tissues, and weakly in heart (*Dr-RNase 1*, *Dr-RNase 2 *and *Dr-RNase 3*; [16]), or brain (*Zf-RNase-1*; [17]). It is important to note that while expression of *RNase Dre1 *and *RNase Dre2 *is clearly detected in several internal organs, RNase activity is not easily detected in "guts" extracts (Figure 1A). Further characterization of these extracts indicated that although RNase activities are present, they are degraded by proteases that seem to have a certain amount of specificity, since most proteins in gut extracts seem unaltered after Coomassie blue staining (Figure 1 and data not shown).

Analysis of expression in whole embryos during developmental stages was also performed (Figure 3B). Strong expression of *RNase Dre1 *was observed during all developmental stages analyzed (2, 5, 12, 24, 72 h after fertilization), with a peak of expression at 72 hours. *RNase Dre2 *was also detected in all developmental stages and peaked at 72 h, although the signal was lower at 5 and 12 h. In contrast, only one of the RNase A homologs, *Zf-RNase-1*, is expressed in early embryo tissues [16,17]. Analysis of RNase activity in embryo extracts showed a pattern consistent with mRNA expression results. Only RNase activities in the 20–30 kDa range were detected, and only in acidic conditions (Figure 3C).

**Figure 4:**
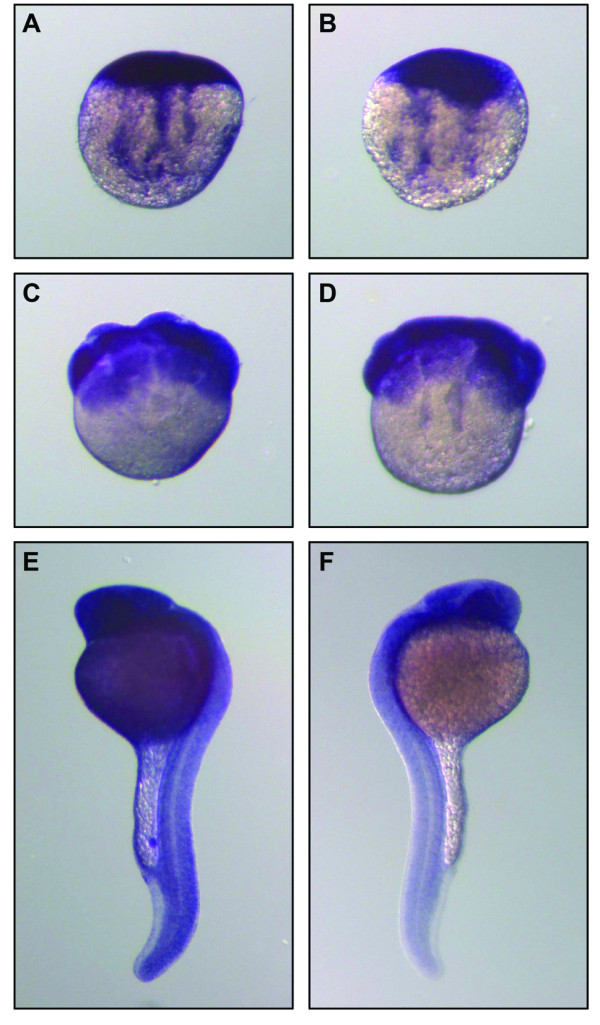
**Localization of *RNase Dre1 *and *RNase Dre2 *expression in zebrafish embryos**. Whole-mount *in situ *hybridization analysis was performed in embryos at the 1-cell stage (A, B), 16-cell stage (C, D) and prim 6 stage (E, F). Left panels, *RNase Dre2 *probe; right panels, *RNase Dre1 *probe.

To further confirm that *RNase Dre1 *and *RNase Dre2 *are expressed in zebrafish embryos, we performed *in situ *hybridization analyses (Figure 4). As expected, both mRNAs were detected in all the embryo stages studied. At the one cell stage (Figure 4, panels A-B) transcripts corresponding to both RNases localized mainly to the animal pole, or the part of the cell that will contribute to the embryo proper. It was also possible to observe RNA projections in structures that resemble cytoskeletal arrangements extending from the animal pole toward the vegetal pole associated with axial streaming of ooplasm [21]. These structures could correspond to RNA being recruited to the embryo from the extraembryonic yolky cytoplasm [22]. At the 16-cell stage (Figure 4, panels C-D) both RNAs gave a strong signal in blastomeres. Twenty six-hour embryos (Figure 4, panels E-F) showed strong expression throughout the embryo, and only *RNase Dre2 *showed weak expression in yolk, with both RNases most highly expressed in eyes.

### RNase T2 enzymes are also expressed in embryos of other organisms

**Figure 5:**
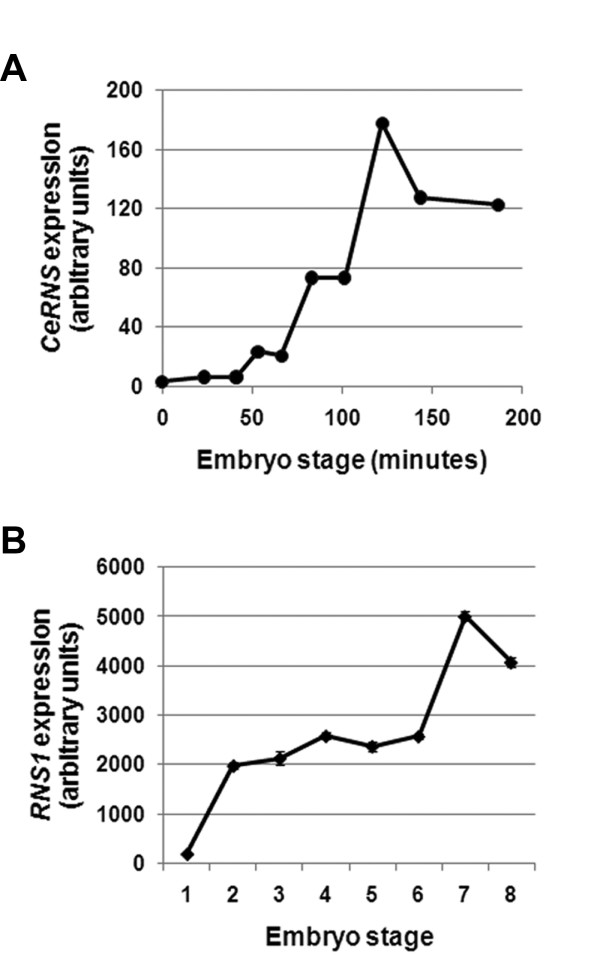
**RNase T2 genes are expressed in embryos in other organisms**. Expression of RNase T2 genes during embryo development in the nematode *Caenorhabditis elegans *(**A**) and the plant *Arabidopsis thaliana *(**B**). Expression data were obtained from public microarray databases. Values indicate arbitrary fluorescence intensity units after normalization. **A**) Stages of nematode embryo development indicated as minutes after fertilization. **B**) Arabidopsis embryo stages: 1, globular; 2, heart; 3, triangle; 4, torpedo; 5, curly cotyledon; 6, curly cotyledon 2; 7, mature cotyledon; 8, green cotyledon.

To investigate whether other RNase T2 homologs also have a role during embryo development we analyzed expression of genes belonging to this family using available microarray data from public databases. *CeRNS*, the only RNase T2 homolog gene in the nematode *Caenorhabditis elegans *genome is also expressed during embryo development (Figure 5A). Microarray data also indicated that *CeRNS *is not expressed in adult tissues (not shown). In situ hybridization results obtained from The Nematode Expression Pattern DataBase (Tadasu Shin-i and Yuji Kohara, unpublished, ) confirmed the expression pattern of *CeRNS *obtained from microarray databases. *CeRNS *embryo expression seems to be ubiquitous, as is the case for *RNase Dre1 *and *RNase Dre2*. In addition, analysis of expression data representing 61 mouse tissues [23] showed that the only RNase T2 homolog present in the mouse genome was also detected in embryonic samples (not shown). Remarkably, embryo expression is not limited to animals. According to microarray data [24], *RNS1*, one of five members of the RNase T2 family present in the plant *Arabidopsis thaliana*, is one of the most highly expressed genes (98–99 percentile) during embryo development (Figure 5B).

### RNase Dre1 represents a gene duplication present only in ray-finned and cartilaginous fishes

**Figure 6:**
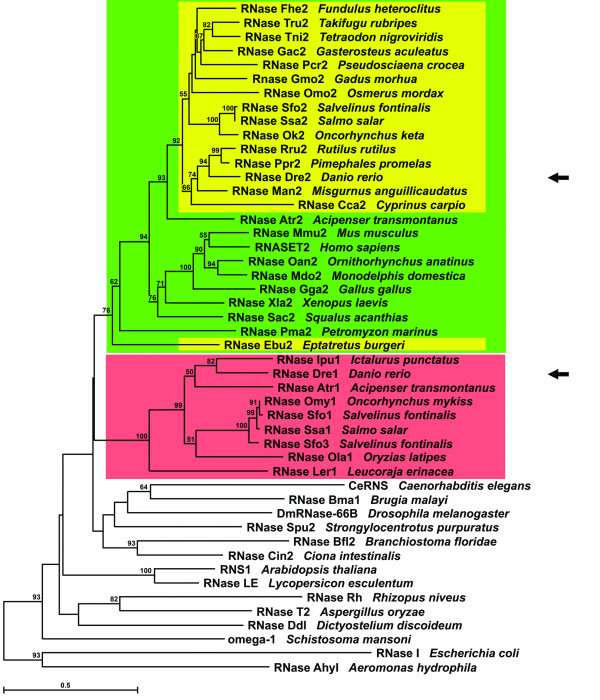
**Phylogenetic relationships of fish RNase T2 proteins, other animal RNase T2 proteins and bacterial, fungal and plant RNase T2s**. Unrooted tree was obtained by the Neighbor-Joining method using only conserved regions. Bootstrap percentages (for 1,000 replications) greater than 50 are shown on interior branches. Color boxes highlight the clades that include fish RNases. Green indicates canonical CAS II, while yellow and red indicate mutations that putatively attenuate RNase activity (see figure 7). RNase Dre1 and RNase Dre2 are indicated with arrows.

In order to understand the evolution of RNase T2 genes in fish and other animals, we searched for sequences belonging to this family in EST and protein databases. We also analyzed the fully sequenced genomes of the ray-finned fishes medaka (*Oryzias latipes*), spotted green pufferfish (*Tetraodon nigroviridis*), and fugu (*Takifugu rubripes*). In these three genomes we identified two genes in each species belonging to the RNase T2 family, as in the zebrafish genome, although only one in each case was also represented in EST collections. Additional sequences belonging to this family were found in EST collections for other fish species, including sharks, lamprey and hagfish (see Figure 6 and Additional File 2 for full list of species).

Among the many RNase T2 sequences available from other organisms, we selected several sequences from vertebrates (platypus, opossum, mouse, human, chicken, frog), an Urochordate, a Cephalochordate, an Echinoderm, nematodes, a trematode and an insect to generate a protein Neighbor-Joining tree of animal RNases (Figure 6). Proteins from plants (RNase LE and RNS1), bacteria (RNase I and RNase AhyI), protozoa (RNase Ddl) and fungi (RNase T2 and RNase Rh) were included to identify the relationship of animal RNases with other proteins in the RNase T2 superfamily.

The tree allowed us to make several inferences on the evolution of the RNase T2 family in animals. Fish RNases cluster in two well defined clades, one represented by RNase Dre1 and the other by RNase Dre2 (red and yellow boxes in Figure 6). Evidence for genes belonging to the two clades was found in all fully sequenced fish genomes by BLAST searches, although only those with EST support were included in the tree shown in Figure 6, because a clear gene model for the other genes was not available. All the fish species for which full genome sequence is available contain only one gene from each clade. Similarly, only one sequence for each clade was found in several fish EST collections with the exception of the brook trout (*Salvelinus fontinalis*), in which a recent duplication gave rise to two copies of the RNase Dre1 homolog (RNase Sfo1 and RNase Sfo3 in Figure 6).

The presence of genes from the two clades in Chondrichthyes (cartilaginous fishes) and Actinopterygii (ray-finned fishes) indicates an ancient origin for the gene duplication that gave rise to these two clades, with the two genes present at least in the last common ancestor of the two classes more than 400 MYA [25]. Analysis of sequence data from earlier Chordata, including sea squirt (*Ciona intestinalis*), amphioxus (*Branchiostoma floridae*), hagfish (*Eptatretus burgeri*), and lamprey (*Petromyzon marinus*) identified only one gene belonging to the RNase T2 family in each species. Since the amphioxus genome has been completely sequenced [26], the presence of only one gene would indicate that the gene duplication occurred after the separation of the Cephalochordata from the main Chordata stem. Since genome coverage for the other species is limited, the exact timing for the gene duplication leading to the two RNase clades cannot be precisely determined. Moderate bootstrap support (76%) for the RNase Dre2 clade suggests that the duplication the predated the divergence of lampreys and hagfishes from jawed vertebrates. However, this result could also mean that the genes in the RNase Dre2 clade conserved more ancestral characteristics than RNase Dre1 after duplication.

Genes belonging to the RNase Dre2 clade were found in all vertebrates analyzed, from hagfish to human. However, RNase Dre1 clade genes were found only in cartilaginous and bony fishes, but not in other vertebrates. Exhaustive analysis of the fully sequenced human and mouse genomes failed to identify RNase Dre1 genes. Interestingly, an RNase T2 pseudogene was found in each of these two genomes (not shown), but in both cases the pseudogene also belonged to the RNase Dre2 clade (the human pseudogene presented 84% identity with the human RNASE T2 protein, while the mouse pseudogene had 63% identity with mouse RNase T2 protein RNase Mmu2). These results suggest that the RNase Dre1 gene was present at least in the last common ancestor of Actinopterygii (ray-finned fishes) and Sarcopterygii (lobe-finned fishes and tetrapods) but was lost in Tetrapods after they diverged.

**Figure 7:**
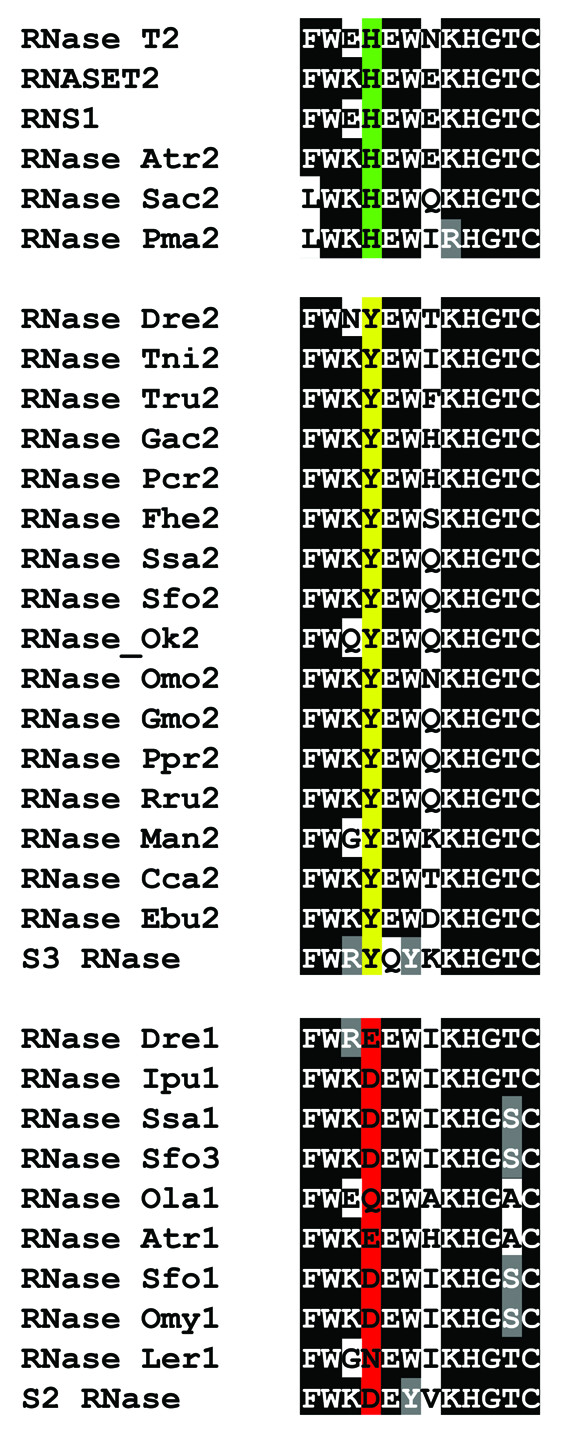
**Mutations in CAS II of fish RNase T2 proteins**. The alignment shows the conserved CAS II region characteristic of RNase T2 enzymes. The absolutely conserved His (black box, white font) is part of the catalytic site of the enzyme. A second His residue (green), possibly involved in substrate binding or stabilization of an intermediate in the catalytic reaction, is mutated in most fish RNases. In the RNase Dre2 clade this His is mutated to Tyr (yellow). In the RNase Dre1 clade the His is mutated to a series of polar amino acids (red). Similar mutations are found in some plant S-RNases (S2 RNase and S3 RNase).

The recent characterization of RNase Ok2 [19] showed that in this protein, a commonly conserved His residue in CAS II (His104 in RNase Rh, Tyr102 in RNase Dre2) is mutated to Tyr. This change most likely affects the catalytic properties of the enzyme and results in lower specific activity [19]. Our results showed that the same mutation is found in all Teleostei (modern ray-finned fishes) (Figure 7), but not in sturgeon (RNase Atr2), sea lamprey (RNase Pma2), and dogfish shark (RNase Sac2), nor in other vertebrates outside fish, suggesting that the mutation appeared and was fixed at the base of this taxon or after the separation of Actinopterygii (ray-finned fishes) and Sarcopterygii (Coelacanths and tetrapods). Interestingly, the same mutation was found in RNase Ebu2 from inshore hagfish, and it is most likely the result of an independent mutation that was fixed in this species (or near taxa).

Remarkably, all analyzed genes belonging to the RNase Dre1 clade also showed mutations in the same position (Figure 7). In this case the canonical His residue characteristic of other RNase T2 enzymes is replaced by a series of charged or polar amino acids: Glu, Asp, Gln, or Asn. Since all genes in this subfamily have substitutions in this position, the loss of the His residue seems to have happened soon after the duplication event that gave rise to the RNase Dre1 clade. Although no mutagenesis experiments have been carried out to show the effect of these mutations, some S-RNases have similar substitutions (Figure 7). S-RNases have low specific activity compared with other RNase T2 enzymes, and such characteristic has been attributed to the lack of this particular His residue [27]; however, they are still active, and this activity is essential for their biological function. Since the amino acid substitutions in this position in S-RNases and RNase Dre1 homologs are the same, it is expected that the changes observed in RNase Dre1 homologs also reduce, but not eliminate, the specific activity of these enzymes.

## Discussion

Ribonucleases from the RNase A and RNase T2 family have been frequently used as models for the study of evolution of gene function. These two types of RNases have similar enzymatic activity and substrate preferences, both being endoribonucleases that mainly hydrolyze bulk single stranded RNA. Both families are also found mainly in extracellular space or associated with the secretory pathway. While the RNase A family is vertebrate specific [16], the RNase T2 family is widespread and members of this family has been found is almost all eukaryotic and many prokaryotic genomes [1,28]. Thus, in spite of this seemingly redundant activity, both enzyme families coexist in vertebrates. Evolution and biological function of RNase T2 proteins have been studied mostly in plants [5-7]; although recent reports of an association of human RNASE T2 with cancer have spiked interest in this protein [29,30].

In this work we characterized the two RNase T2 genes present in the zebrafish genome. A recent analysis of RNase A genes from this fish suggests that the available genome sequence may not be complete, as at least one RNase A gene found in cDNA libraries is not found in the genome [15,16,31]. However, based on the lack of any other RNase T2 sequence in zebrafish cDNA collections, the presence of only two genes in the other fully sequenced fish genomes, and our phylogenetic analysis, we feel confident that only two RNase T2 genes are present in the zebrafish genome.

We were able to detect ribonuclease activities in zebrafish extracts that show the molecular weight and enzymatic properties expected for the proteins encoded by *RNase Dre1 *and *RNase Dre2*. In addition, the conservation of the active site residues, and the high sequence similarity between the zebrafish RNases and RNase Ok2 (more than 50% identity between RNase Dre2 and RNase Ok2), which was shown to be an active ribonuclease by purification from salmon liver [19] strongly suggest that RNase Dre1 and RNase Dre2 are active ribonucleases.

Fish seem unusual among animals because in all species analyzed there were two genes belonging to the RNase T2 family, unlike all other animals in which only one gene has been found. Genomic data indicate that a whole genome duplication event (WGD) occurred in the fish lineage after the separation of teleosts from the main tetrapod stem. This WGD explains the occurrence of many ray-finned fish-specific gene duplications [32,33]. However, this WGD is proposed to have occurred after the separation of the Acipenseriformes and the Semionotiformes from the lineage leading to teleost fish, but before the divergence of Osteoglossiformes [32]. Thus, the gene duplication event that gave rise to both RNase T2 genes present in fish genomes cannot correspond to this ray-finned fish-specific WGD, since genes corresponding to the RNase Dre1 and RNase Dre2 clades were found in sturgeons (ray-finned fishes but not Teleostei) and sharks (Chondrichthyes).

**Figure 8:**
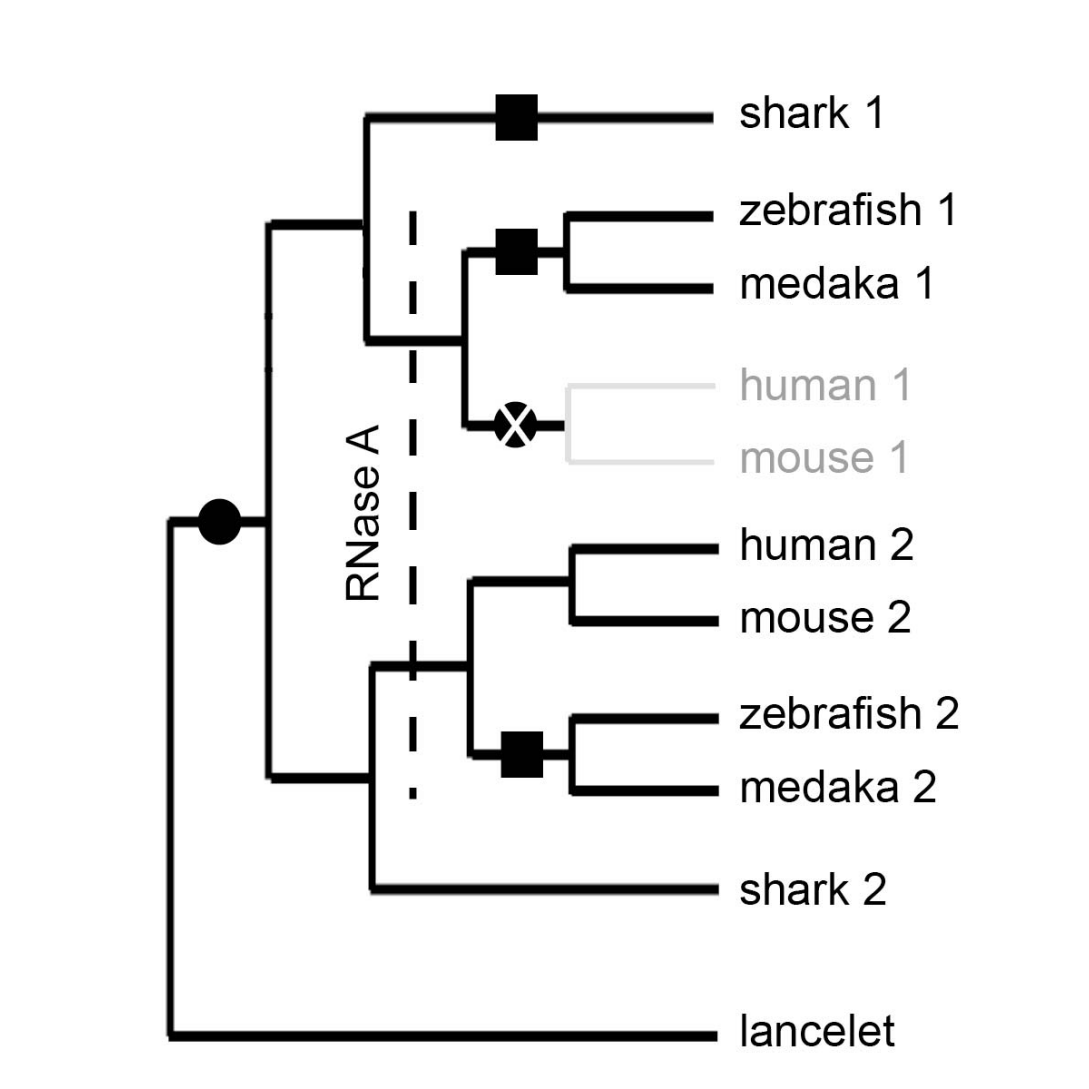
**Hypothetical model of RNase T2 evolution in animals**. An ancestral RNase T2 gene present in the last common ancestor of lancelet and higher chordate was duplicated after the separation of these two groups, but before the separation of Chondrichthyes and Teleostomi (black circle). Sometime after this duplication event RNase A genes emerged, most likely after the separation of Chondrichthyes and Teleostomi. The presence of RNase A could have released some selective pressure on RNase T2 genes, allowing the fixation of mutations in the active site conserved region (squares, H/Y/E-D position in CAS II), and the disappearance of one of the genes in tetrapods (black circle with white X).

In contrast, the lack of RNase Dre1 orthologs in all tetrapods indicates that this gene was lost in this lineage soon after the separation from the Actinopterigii. Moreover, any duplicated gene produced by the WGD that occurred in the fish lineage was also lost. Interestingly, according to Cho and Zhang [16], RNase A genes may have appeared in the chordate lineage in the last common ancestor of these two groups. While the success of this new gene family was mixed in ray-finned fish (zebrafish has 4 RNase A genes, but fugu and Tetraodon seem to have none), it has been successfully maintained and underwent a large diversification in tetrapods. In plants, where no RNase A genes exist, RNase T2 genes have radiated and diversified to a greater extent, in a way similar to that observed for the RNase A family in animals (A. Meyer and G. MacIntosh, unpublished). Thus, it is tempting to hypothesize that the presence of RNase A genes influenced the evolution of the RNase T2 family in ray-finned fish and tetrapods (Figure 8).

This hypothesis could be supported by a series of observations. Plant RNase T2 genes and vertebrate RNase A genes show patterns of gene sorting [9,16](A. Meyer and G. MacIntosh, unpublished) such as the presence of different gene numbers in different species and the lack of clear orthologs among species. In contrast, vertebrate RNase T2 genes form two monophyletic groups, one exclusive to fish, and the other including all vertebrates (Figure 6).

The functions acquired by duplicated genes after extensive radiation seem to be similar for the two types of enzymes in plants and animals; i.e., the biological roles assigned to many RNase A proteins in animals are similar to those of RNase T2 proteins in plants. For example, several members of the RNase A family have antimicrobial properties. Eosinophil associated RNases have antiviral (RNase 2 and RNase 3 in humans) and antibacterial (RNase 3) function, and angiogenin and RNase 7 have antibacterial and antifungal activities[14]. Similarly, plant RNase T2 proteins inhibit hyphal elongation of the pathogenic oomycete *Phytophthora parasitica *[34], and are induced by viral and fungal pathogens [35,36]. In addition, both animal RNase A and plant RNase T2 enzymes have cytotoxic properties, for example frog oocyte RNases used as anticancer drugs [37], and flower S-RNases that reject pollen during self-incompatible pollination [38]. The cytotoxic properties of these enzymes are probably as a consequence of their role in defense, as in the case of frog oocyte RNases [39], or have evolved from a defensive role, as in the case of S-RNases [7]. A nutritional role has also been proposed for plant RNase T2 and animal RNase A enzymes. RNase A is secreted into the mammalian intestine where it helps digest RNA from gut bacteria to recover nutrients [12]; while expression of plant RNase T2 enzymes is induced when phosphate in the soil is limited [40-42]as part of a phosphate scavenging system [43].

Finally, most plant RNase T2 enzymes and vertebrate RNase A enzymes show strong tissue specificity and lack of expression in early embryos, suggesting that they are involved in immune and stress responses rather than having a housekeeping role [16]. On the other hand, animal RNase T2 enzymes, and a few plant ones like Arabidopsis RNS2 [40] (A. Meyer and G. MacIntosh, unpublished), seem to be constitutively expressed, suggesting that they could have a housekeeping function. This function, conserved through evolution, could be responsible for the conservation of the RNase T2 family in animals, in spite of the presence of RNase A.

We hypothesize that the role of RNase T2 enzymes could be to recycle bulk RNA (mostly rRNA) throughout the life of the cell, and not only in times of nutrient deprivation as has been proposed before. RNA is an important source of P and N, and turnover of this molecule should be important for P and N homeostasis. Accordingly, some RNase T2 enzymes have been found in intracellular compartments, supporting the idea of a role recycling RNA in normal cells. For example, human RNASET2 has been found to accumulate in the lysosome [44], while Arabidopsis RNS2 is present in intracellular fractions, probably associated to the vacuole or the ER [45]. In the case of zebrafish RNase Dre1 and RNase Dre2 the localization is unknown, but subcellular localization predictions using different programs (see Material and Methods) indicate either extracellular or microsomal/lysosomal localization, almost identical to predictions for the human enzyme. It is interesting to note that the alternative splicing observed for RNase Dre1 alters the protein's signal peptide, opening the possibility that this protein localizes to different subcellular compartments. Alternative processing resulting in different subcellular localizations has already been described for tomato ribonucleases [46,47].

The high level of expression of RNase T2 enzymes in embryonic tissues is also notable. This pattern could also be a consequence of the proposed housekeeping role for RNase T2s. The high metabolic activity of embryos could demand a high level of RNase activity to process cellular material as it is being renewed. Alternatively, we could look for other explanations for the embryonic role of RNase T2 enzymes. A highly speculative but attractive idea is that secreted RNases can control the activity of small RNAs [48].

Following this rationale, we could speculate that the embryonic role of RNase T2 enzymes is to shield embryonic tissues from unwanted small RNAs. In plants, RNA silencing is reset in each generation [49]. This property of silencing was shown for virus induced gene silencing (VIGS) and posttranscriptional gene silencing (PTGS). Arabidopsis *RNS1 *is one of the most highly expressed genes during all the stages of embryo development. Importantly, RNS1 is secreted to the apoplastic space [45]. It has been shown that the outer integument of the developing seed can provide a symplastic route for transport from maternal tissues to the developing seed, but the transfer between the outer integument and the inner integument and between the integument and the embryo are apoplastic [50,51]. Thus, any RNA signal would have to travel through the apoplast to reach the embryo would find a barrier due to accumulation of RNS1. In the nematode *Caenorhabditis elegans *it has been shown that phenotypes induced by RNAi can last only for two or three generations [52], and only a small subset of genes (13/171) that could be inheritably silenced for longer periods of time [53]. These results suggest that transmission of silencing from maternal to embryonic tissues could be regulated also in animals. In this context, secreted RNases would form a RNA surveillance field [54], that stops the spreading of small RNAs.

In summary, it seems possible that the emergence of RNase A affected the evolution of RNase T2 proteins in animals. The smaller size of RNase A proteins, which could be more energetically favorable, could favor the use of this protein instead of RNase T2 proteins for defense roles in animals. However, RNase T2 proteins have not been completely replaced in animals, most likely because they also have a housekeeping function in an intracellular compartment that cannot be carried out by RNase A.

## Conclusion

The zebrafish genome contains two RNase T2 genes, *RNase Dre1 *and *RNase Dre2*. These genes are part of two phylogenetic clades, one conserved in all chordates (the RNase Dre2 clade), and another fish-specific (the RNase Dre1 clade). Expression analyses indicate that *RNase Dre2 *is present in all tissues and developmental stages in zebrafish, suggesting a housekeeping role for these enzymes. This idea is further supported by the conservation of RNase T2 genes in all the genomes analyzed. Analyses of the evolution of the RNase T2 family in animals, and comparisons with the evolution of RNase T2 in plants and RNase A in vertebrates suggest that the emergence of RNase A affected the evolution of RNase T2 proteins in animals.

## Methods

### Database searches and sequence identifications

Identification of RNase T2 genes was done by BLAST searches in the zebrafish (*Danio rerio*) genome (version Zv7, available through the National Center for Biotechnology Information (NCBI), . Analyses of the medaka (*Oryzias latipes*), spotted green pufferfish (*Tetraodon nigroviridis*), and other full genomes was also performed using NCBI resources. Analysis of the fugu (*Takifugu rubripes*) genome was performed using Assembly Release 4 from the Fugu BLAST server . Expressed sequence tag (EST) sequences and protein sequences were also obtained by BLAST searches of the NCBI EST-other and non-redundant databases respectively. Analysis of genome organization for the RNase Dre1 and RNase Dre2 genes was done using contigs obtained by combining information from cDNAs cloned by our laboratory (supplementary dataset) and ESTs obtained from NCBI EST-other.

Prediction of signal peptides and subcellular localization was carried out using PSORT [55], WoLF PSORT [56] and SignalP and TargetP [57].

Arabidopsis microarray data were obtained from the Arabidopsis information Resource (TAIR) database. Mouse microarray data were obtained from the Genomics Institute of the Novartis Research Foundation SymAtlas . Nematode microarray data were obtained from WormBase . In all cases, normalized data were used, and values belonging to the same experiment set were compared.

### Zebrafish samples preparation and cDNA cloning and RT-PCR

Wild zebrafish and laboratory strain WIC were used in our experiments. *RNase Dre1 *and *RNase Dre2 *cDNAs were amplified from 48 hr post-fertilization embryo RNA using primers designed based on database sequences. Embryos were broken by forcing the sacs through a syringe fitted with a sterile needle prior to extraction. Total RNA was purified using the TRIzol reagent according to the manufactures directions (Invitrogen), and cDNAs were synthesized using the iSCRIPT kit (BioRAD). PCR was performed using the following primers: *RNase Dre1*F 5'CGCGATATCACAGGCTGTTTGTTACTGAC3', *RNase Dre1*R 5'CGCCCATGGGCGCTTGCACCGGTGGGTAATA3', *RNase Dre2*F 5'CGCGATATCACAGACTCTCAGAACAGACG3' and *RNase Dre2*R 5'CGCCCATGGGGTTACATGGCTCATGAGGA3'. During cloning, we amplified two PCR products corresponding to *RNase Dre1*. Both bands from *RNase Dre1 *and a single band from *RNase Dre2 *amplification were gel purified using a Gel Purification kit (Promega). The genes were cloned using the pGEM-T Easy kit (Promega) and sequenced. Sequencing reactions were performed at the DNA Facility at Iowa State University using T7 and SP6 primers.

Expression analyses were performed using semiquantitative RT-PCR. Adult fish were dissected into the following organs: brain, eyes, heart, liver, gut (digestive system), muscle, ovary, testis, and skin. Excluding reproductive organs, all tissue samples came from fish of both sexes. RNA was extracted and cDNA was generated as described previously. PCR amplification was done using GoTAQ Green Master Mix (Promega). The cDNA corresponding to the ribosomal protein p70 was used as loading control for RT-PCR. The primer sequences used for p70 were p70/6sk-r1 5'AGCTTGCCGCCCGTCTGAAA3', and p70/6sk-f1 5'CATGGCGACGGTGCGTTCAT3'. Primer sequences for *RNase Dre1 *and *RNase Dre2 *are the same as those listed above. Gels were stained with ethidium bromide and visualized using the NIH Image program. All experiments were performed a minimum of 3 times and a representative sample was chosen for each figure.

### RNase Activity

Adult fish of both sexes were dissected into the following sections: head, including all tissues above the heart; body, including skin, muscle, and bones of the main body; and gut, including the digestive and reproductive systems. Proteins were extracted from each section following the method used by MacIntosh *et al*. [58]. The protein extraction protocol was modified by eliminating β-mercaptoethanol and polyvinylpolypyrrolidone from the extraction buffer. Protein was quantified according to the Bradford method. RNase activity was determined by an *in gel *assay according to Yen and Green [59], using high molecular weight RNA purified from commercial torula (yeast) RNA (SIGMA). One hundred μg of protein were run for each sample. Gels were incubated in 0.1 M Tris-HCl at either pH 6.0 or pH 7.0 as identified in the figure. In parallel, SDS-PAGE gels were run using 100 μg of protein to verify loading amounts and protein quality. All experiments were performed a minimum of 3 times and a representative sample was chosen for each figure.

### *In situ *hybridizations

Whole-mount in situ hybridizations were performed as described by Essner *et al*. [60], using 1-cell (~30 min. post fertilization), 16-cell (~1.5 h post fertilization) and prim 6 (~26 h post fertilization) embryos [61]. RNA probes were prepared *in vitro *transcription from linearized templates of *RNaseDre1 *and *RNaseDre2 *cDNA in the pGEM T-Easy vector.

### Phylogenetic analysis

Protein sequences were aligned using ClustalW2 [62] followed by manual adjustments. PAUP 4.0 software [63] was used for phylogenetic analyses. Phylogenetic trees were constructed using the Neighbor-Joining tree method [64] with 1,000 bootstrap replications.

## Authors' contributions

MSH carried out the gene expression analyses, RNase activity characterization, and part of the cloning. LR and AU performed the initial cloning and characterization of alternative splicing. YW and JJE carried out the in situ hybridizations. GCM conceived of the study, and performed the phylogenetic analyses. MSH and GCM participated in the design of the study and drafted the manuscript. All authors read and approved the final manuscript.

## Supplementary Material

Additional file 1 **Supplemental Dataset**. mRNA and predicted protein sequences of zebrafish RNase T2 genes Click here for file

Additional file 2 **Supplemental Table 1**. RNase T2 proteins used for phylogenetic analysis Click here for file
