# Supplementary material for: Zebrafish RNase T2 genes and the evolution of secretory ribonucleases in animals
Source: BMC Evol Biol. 2009 Jul 20;9:170. doi: 10.1186/1471-2148-9-170 (PMC2720953; doi:10.1186/1471-2148-9-170)
Supplement: Additional file 1 — Supplemental Dataset. mRNA and predicted protein sequences of zebrafish RNase T2 genes [file 1471-2148-9-170-S1.pdf]

Supplemental Dataset: mRNA and protein sequences of zebrafish RNase T2 genes

RNase Dre1 short mRNA

>RNase_Dre1a
GATATCACAGACTCTCAGAACCCACTGATCAGCATGACCTACAATAAACACATTCTCTGG
GCCTTTACCGCTGCTCTGGCCACAGGTTGGGTCCTTAGTAATGATGAAGGATGCTATTAT
GGGACTGTCATGAAGCACTCATGCAACTGGACTTGTATGCTGCTCACTCTTCAATGGCCT
GGAAGTTTCTGTATAGGTCTTACTAATAAAACGATTTGCAAAATACCACTGACTATTCAA
AACTGGACCATCCATGGCCTATGGCCGATGCATACAGGTCACTGCTGTAACTGTTGGCCA
ATATTTCATTCCCACCTTCAGGAAATCGAGCCAGAACTCACTCAGCTGTGGCCATCTTTA
ATAAAAGGAAAGCATTTCTTCAACTTTTGGAGGGAGGAATGGATTAAACACGGGACGTGT
GCTGGCTGTGACGGGGCCATGGGTTCACCGCTTCTTTACTTCCAGGCTGCAGTCAAGCTT
CGAAAACTCTTTGACATTAATAGCGTCTTGGAAAGCTCTGGAATCAAAGCCTCATGTGAG
GTGTCCTATAAGTATGACGACATAAGCAAGGCCCTGACCTCACTGCTGGGAGACAACTTT
GATTTGCAATGTGTGACTGACAGTAAGGGTCGTGAAGCATGGATCCAGCTGAAGATCCAT
CTTTCGAGGAACCAAACCATTGGATGCCCCACAGAGAAGCAAGACGAAGCATTCTATAAT
TTAGCGTGGTATAAAAGCCCTGGACATCCTTGTCCGAAGAACACCACCATCTTTTTTGTC
CCAATTAACTATGACAATCCTCATGAGCCATGT

RNase Dre1 protein encoded by short mRNA

>RNase_Dre1a
MTYNKHILWAFTAALATGWVLSNDEGCYYGTVMKHSCNWTCMLLTLQWPGSFCIGLTNKT
ICKIPLTIQNWTIHGLWPMHTGHCCNCWPIFHSHLQEIEPELTQLWPSLIKGKHFFNFWR
EEWIKHGTCAGCDGAMGSPLLYFQAAVKLRKLFDINSVLESSGIKASCEVSYKYDDISKA
LTSLLGDNFDLQCVTDSKGREAWIQLKIHLSRNQTIGCPTEKQDEAFYNLAWYKSPGHPC
PKNTTIFFVPINYDNPHEPC

RNase Dre1 long mRNA

>RNase_Dre1b
GATATCACAGACTCTCAGAACAGACGGATCAGCATGACCTACAGTAAGAATCTACCTGAA
TAGCTATATAACCTCTGTTTAAATGCATATTTACTGTAACAATCTGTATTGTTTTAGATA
AACACATTCTCTGGGCCTTTACCGCTGCTCTGGCCACAGGCTGGGTCCTTAGTAATGATG
AAGGATGCTATTATGGGACTGTCATGAAGCACTCATGCAACTGGACTTGTATGCTGCTCA
CTCTTCAATGGCCTGGAAGTTTCTGTATAGGTCTTACTAATAAAACGATTTGCAAAATAC
CACTGACTATTCAAAACTGGACCATCCATGGCCTATGGCCGATGCATACAGGTCACTGCT
GTAACTGTTGGCCAATATTTCATTCCCACCTTCAGGAAATCGAGCCAGAACTCACTCAGC
TGTGGCCATCTTTAATAAAAGGAAAGCATTTCTTCAACTTTTGGAGGGAGGAATGGATTA
AACACGGGACGTGTGCTGGCTGTGACGGGGCCATGGGTTCACCGCTTCTTTACTTCCAGG
CTGCAGTCAAGCTTCGAAAACTCTTTGACATTAATAGCGTCTTGGAAAGCTCTGGAATCA
AAGCCTCATGTGAGGTGTCCTATAAGTATGACGACATAAGCAAGGCCCTGACCTCACTGC
TGGGAGACAACTTTGATTTGCAATGTGTGACTGACAGTAAGGGTCGTGAAGCATGGATCC
AGCTGAAGATCCATCTTTCGAGGAACCAAACCATTGGATGCCCCACAGAGAAGCAAGACG
AAGCATTCTATAATTTAGCGGCGTATAAAAGCCCTGGACATCCTTGTCCGAAGAACACCA
CCATCTTTTTTGTCCCAATTAACTATGACAATCCTCATGAGCCATGTAAC

RNase Dre1 protein encoded by long mRNA

>RNase_Dre1b

MKHSCNWTCMLLTLQWPGSFCIGLTNKTICKIPLTIQNWTIHGLWPMHTGHCCNCWPIFH
SHLQEIEPELTQLWPSLIKGKHFFNFWREEWIKHGTCAGCDGAMGSPLLYFQAAVKLRKL
FDINSVLESSGIKASCEVSYKYDDISKALTSLLGDNFDLQCVTDSKGREAWIQLKIHLSR
NQTIGCPTEKQDEAFYNLAAYKSPGHPCPKNTTIFFVPINYDNPHEPCN

RNase Dre2 mRNA

>RNase_Dre2
ACAGGCTGTTTGTTACTGACAGGAAAGGAAGTGGCTTTAAAGTGACTCAACCGCACATCA
TTTTTTTAAAGAAAGTCTGGGCAGATCTACTTCAGTCAGAGTTATACTAATAGGACGAAA
GGAGAACTGTAACGTTATGTGAATTGGAGCGGCACTTCAGACAATAACAGATTAACTGTT
TTACTCTACCTGGAAACATGAGATTCATTGCATTTGCTGTCATCTTTAGTGCTGTATATC
TTTGCTCATCAGCCTTTACCCATCCTCGGGGAGAATGGACAAAACTTATACTGACCCAGC
ATTGGCCACAGACATTTTGCAAAATGGAACACTGCAAAACAGATTTCAGCTATTGGACTC
TGCATGGATTATGGCCCAACACTGGTGTAAGGTGCAACACATCTTGGCATTTTAATGCCA
GTTTGATTGAGGACATACTACCAGAAATGGAGAAATTCTGGCCAGATCTGCTAGAACCAT
CTTCCCCAAAATTTTGGAATTATGAATGGACGAAACACGGGACCTGTGCTGCAAAATCAG
AGTCTTTAAACAGTGAACATAAGTACTTTGGCAAAGCTCTCGAACTCTACCACAAGTTTG
ACCTTAACAGTGTTTTGCTGAAGAATCAAATTGTGCCCTCTGAGAAGCATTACACGCTGG
AGGATGTGGAGGAAGCCATTACAAGTGCCTACGGAGTAAAGCCCAAGATCCAGTGTGTCC
ACCCAGGACAGGGAGGCCAGGTTCAAATTTTGGGCCAAATAGAGATCTGTGTTGACAGGG
ATTTCCAACTGATGGGTTGTGAAAAGTCCAGCGAAGACACCTGGAGCAATGACCTCCCCA
CAGTGCCTGTCAGTGGCCAGTCAGGACTCAGCGTGTGTGATCACTCCATGCCAGTCTATT
ACCCACCGGTGCAAGCG

RNase Dre2 protein

>RNase_Dre2
MRFIAFAVIFSAVYLCSSAFTHPRGEWTKLILTQHWPQTFCKMEHCKTDFSYWTLHGLWP
NTGVRCNTSWHFNASLIEDILPEMEKFWPDLLEPSSPKFWNYEWTKHGTCAAKSESLNSE
HKYFGKALELYHKFDLNSVLLKNQIVPSEKHYTLEDVEEAITSAYGVKPKIQCVHPGQGG
QVQILGQIEICVDRDFQLMGCEKSSEDTWSNDLPTVPVSGQSGLSVCDHSMPVYYPPVQA
