# Supplementary material for: Zebrafish RNase T2 genes and the evolution of secretory ribonucleases in animals
Source: BMC Evol Biol. 2009 Jul 20;9:170. doi: 10.1186/1471-2148-9-170 (PMC2720953; doi:10.1186/1471-2148-9-170)
Supplement: Additional file 2 — Supplemental Table 1. RNase T2 proteins used for phylogenetic analysis [file 1471-2148-9-170-S2.pdf]

Supplementary table 1. RNase T2 proteins used for phylogenetic analysis

Name	Accession #	species	common name	CASII	source
RNase T2	P10281	*Aspergillus oryzae*	Koji-kin	H	Protein
RNase Rh	P08056	*Rhizopus niveus*		H	Protein
RNase DdI	XP_640939	*Dictyostelium discoideum*		H	Protein
RNase LE	P80022	*Lycopersicon esculentum*	tomato	H	Protein
RNS1	P42813	*Arabidopsis thaliana*	thale-cress	H	Protein
omega-1	ABB73002	*Schistosoma mansoni*		H	Protein
RNase Ler1	CV222118	*Leucoraja erinacea*	little skate	N	EST
RNase Atr1	DR976433	*Acipenser transmontanus*	American sturgeon	E	EST
RNase Ola1	BJ501340	*Oryzias latipes*	medaka	Q	EST
**RNase Dre1**	**FJ460211**	***Danio rerio***	**zebrafish**	**E**	**this work**
RNase Ipu1	CV994888	*Ictalurus punctatus*	channel catfish	D	EST
RNase Sfo3	EV387897	*Salvelinus fontinalis*	brook trout	D	EST
RNase Ssa1	DW561904	*Salmo salar*	Atlantic salmon	D	EST
RNase Sfo1	EV393106	*Salvelinus fontinalis*	brook trout	D	EST
RNase Omy1	CA371032	*Oncorhynchus mykiss*	rainbow trout	D	EST
RNase Spu2	XP_780287	*Strongylocentrotus purpuratus*	purple sea urchin	H	Protein
RNase Ebu2	BJ653399	*Eptatretus burgeri*	inshore hagfish	Y	EST
RNase Pma2	CO553054 + CO552166	*Petromyzon marinus*	sea lamprey	H	EST
RNase Atr2	DR976045	*Acipenser transmontanus*	American sturgeon	H	EST
RNase Cca2	EX822236	*Cyprinus carpio*	common carp	Y	EST
RNase Fhe2	DR441730	*Fundulus heteroclitus*	killifish	Y	EST
RNase Omo2	EL528012	*Osmerus mordax*	rainbow smelt	Y	EST
Rnase Gmo2	EX741865	*Gadus morhua*	Atlantic cod	Y	EST
RNase Tni2	CAG05697	*Tetraodon nigroviridis*	spotted green pufferfish	Y	Protein
RNase Tru2	CA330011	*Takifugu rubripes*	fugu	Y	EST
RNase Gac2	DW635779	*Gasterosteus aculeatus*	three-spined stickleback	Y	EST
RNase Pcr2	CX349007	*Pseudosciaena crocea*	large yellow croaker	Y	EST
RNase Ok2	BAB55596	*Oncorhynchus keta*	chum salmon	Y	Protein
RNase Ssa2	EG914237	*Salmo salar*	Atlantic salmon	Y	EST
RNase Sfo2	EV393679	*Salvelinus fontinalis*	brook trout	Y	EST
RNase Man2	BJ820572	*Misgurnus anguillicaudatus*	oriental weatherfish	Y	EST
**RNase Dre2**	**FJ460212**	***Danio rerio***	**zebrafish**	**Y**	**this work**
RNase Ppr2	DT189242	*Pimephales promelas*	fathead minnow	Y	EST
RNase Rru2	EG548700	*Rutilus rutilus*	roach minnow	Y	EST
RNase Xla2	NP_001086583	*Xenopus laevis*	African clawed frog	H	Protein
RNase Sac2	EG027303	*Squalus acanthias*	Dogfish Shark	H	EST
RNase Gga2	NP_001034580	*Gallus gallus*	chicken	H	Protein
RNASET2	NP_003721	*Homo sapiens*	human	H	Protein
RNase Mmu2	NP_001077407	*Mus musculus*	mouse	H	Protein
RNase Mdo2	XP_001381570	*Monodelphis domestica*	gray short-tailed opossum	H	Protein
RNase Oan2	EY201560	*Ornithorhynchus anatinus*	duck-billed platypus	H	EST
RNase Cin2	BW482808	*Ciona intestinalis*	Sea squirt	H	EST
RNase Bfl2	BW708730	*Branchiostoma floridae*	Florida lancelet (Amphioxus)	H	EST
CeRNS	NP_503370	*Caenorhabditis elegans*	nematode	H	Protein
RNase Bma1	EDP29148	*Brugia malayi*	filarial nematode	H	Protein
DmRNase-66B	CAA52884	*Drosophila melanogaster*	fruit fly	H	Protein
RNase AhyI	CAA47438	*Aeromonas hydrophila*		H	Protein
RNase I	NP_752630	*Escherichia coli*		Y	Protein
